# Secreted Aspartyl Proteinases Targeted Multi-Epitope Vaccine Design for *Candida dubliniensis* Using Immunoinformatics

**DOI:** 10.3390/vaccines11020364

**Published:** 2023-02-05

**Authors:** Nahid Akhtar, Jorge Samuel Leon Magdaleno, Suryakant Ranjan, Atif Khurshid Wani, Ravneet Kaur Grewal, Romina Oliva, Abdul Rajjak Shaikh, Luigi Cavallo, Mohit Chawla

**Affiliations:** 1 Department of Research and Innovation, STEMskills Research and Education Lab Private Limited, Faridabad 121002, India; 2 Physical Sciences and Engineering Division, Kaust Catalysis Center, King Abdullah University of Science and Technology (KAUST), Thuwal 23955-6900, Saudi Arabia; 3 School of Bio-Engineering and Bio-Sciences, Lovely Professional University, Phagwara 144411, India; 4 Department of Sciences and Technologies, University Parthenope of Naples, Centro Direzionale Isola C4, 80143 Naples, Italy

**Keywords:** *Candida dubliniensis*, candidiasis, immunoinformatics, molecular docking, molecular dynamic simulations, multi-epitope vaccine

## Abstract

*Candida dubliniensis* is an opportunistic pathogen associated with oral and invasive fungal infections in immune-compromised individuals. Furthermore, the emergence of *C. dubliniensis* antifungal drug resistance could exacerbate its treatment. Hence, in this study a multi-epitope vaccine candidate has been designed using an immunoinformatics approach by targeting *C. dubliniensis* secreted aspartyl proteinases (SAP) proteins. In silico tools have been utilized to predict epitopes and determine their allergic potential, antigenic potential, toxicity, and potential to elicit interleukin-2 (IL2), interleukin-4 (IL4), and IFN-γ. Using the computational tools, eight epitopes have been predicted that were then linked with adjuvants for final vaccine candidate development. Computational immune simulation has depicted that the immunogen designed emerges as a strong immunogenic candidate for a vaccine. Further, molecular docking and molecular dynamics simulation analyses revealed stable interactions between the vaccine candidate and the human toll-like receptor 5 (TLR5). Finally, immune simulations corroborated the promising candidature of the designed vaccine, thus calling for further in vivo investigation.

## 1. Introduction

*Candida dubliniensis* (*C. dubliniensis*) is an opportunistic fungal pathogen which was identified for the first time in Dublin in 1995 from the oral cavity of HIV-infected individuals; and since then the pathogen has been reported globally at the prevalence rate of 0.5–7.0% [1,2,3,4]. *C. dubliniensis* is found correlated with fungal pneumonia, chronic meningitis, spondylodiscitis, tricuspid valve endocarditis, oral lichen planus, and denture stomatitis in immunocompromised patients [5,6,7,8,9,10,11]. Moreover, *C. dubliniensis* has been associated with oral candidiasis in diabetic patients undergoing insulin treatment [12]. Recently, *C. dubliniensis* has been reported in SARS-CoV-2 infected patients [13]. The increased clinical awareness of *C. dubliniensis* is attributed due to antifungal drug resistance and reduced susceptibility to antifungal drugs; for instance, fluconazole, flucytosine, voriconazole, and amphotericin B [1,7,14].

Since *C. dubliniensis* has been associated with invasive candidiasis exhibiting antifungal drug resistance, it is of the utmost necessity to look for novel methods to control and treat the infection. Keeping this objective in mind, a novel *C. dubliniensis* vaccine candidate has been designed using an immunoinformatics approach in the current study. 

In order to design the vaccine candidate for *C. dubliniensis,* secreted aspartyl protease (SAP) proteins were chosen since they have been found to be correlated with virulence of various pathogenic fungi belonging to the *Candida* genus, viz. *Candida tropicalis*, *Candida albicans*, and *Candida parapsilosis* [15]. In *C. albicans*, 10 SAP proteins (SAP1-10) have been reported, while in *C. dubliniensis* 8 SAP proteins are found [16]. *C. dubliniensis* lacks orthologs of the *Candida albicans* SAP4 and SAP5 [14,17]. SAPs aid in fungal adhesion and invasion of host tissue by damaging the host extracellular matrix, breaking down cellular proteins, and disintegrating the cell membrane [18]. In *C. dubliniensis*, SAPs act as virulence factors and help in the nutrient acquisition and nitrogen metabolism [16]. Moreover, *C. dubliniensis* SAPs could be associated with invasive candidiasis and hypha formation [16]. Previously, SAPs have been targeted against *C. albicans* and *C. tropicalis* for vaccine development [19,20,21,22,23]. Similarly, in another study, *C. tropicalis* SAP2 was used to develop vaccines for *C. tropicalis* using an immunoinformatics approach [24]. Moreover, the intranasal and intravaginal administration of SAP from *C. albicans* with cholera toxin as an adjuvant elicited the production of anti-SAP antibodies and protected rats from vaginal candidiasis [19]. A recombinant *C. albicans* SAP2 protein, rSAP2t, generated anti-rSAP2t IgA and IgG immunoglobulins and protected rats from *C. albicans* infection [21]. Similarly, immunization with another vaccine candidate, P120, consisting in a recombinant *C. parapsilosis* SAP2 protein in alum adjuvant, produced SAP2 specific antibodies and increased levels of interleukin-4, interleukin-17, and interferon-γ in mice models [25]. Furthermore, the P120 vaccine increased the survival of mice during *C. tropicalis*-based systemic candidiasis by activating the humoral and cellular immune responses [25]. Moreover, the sera from mice immunized with *C. parapsilosis* SAP2 protein have also been reported to inhibit *C. tropicalis* biofilms [23]. Since SAPs (*Candida)*-based vaccines have been reported to develop protection against fungal infections in different animal models, *C. dubliniensis* SAPs appear promising targets for vaccine candidates in *C. dubliniensis*-mediated infections. The proteins SAPs from *C. dubliniensis* were used to identify antigenic B-cell and T-cell epitopes that could be used for vaccine construct development using computational tools. Next, the properties of the epitopes, such as allergic potential, antigenic potential, toxicity, and potential to elicit interleukin-2 (IL2), interleukin-4 (IL4), and IFN-γ were predicted. Finally, the best epitopes were linked together with adjuvants to formulate a vaccine candidate against *C. dubliniensis*. Afterwards, the tertiary structure of the vaccine candidate was predicted and molecular docking and molecular dynamics studies were performed in order to determine the interaction of the vaccine candidate with the immune cells.

## 2. Materials and Methods

### 2.1. Recovery and Analysis of SAPs for C. dubliniensis

Sequences of *C. dubliniensis* SAPs, namely SAP1, SAP2, SAP3, SAP6, SAP7, SAP8, SAP9, and SAP10, were obtained from GenBank. The accession numbers of the *C. dubliniensis* SAPs are listed in Table 1. *C. dubliniensis* SAPs’ antigenicity was determined by the Vaxijen 2.0 online server [26]. 

### 2.2. Designing a Vaccine Candidate

#### 2.2.1. Predicting Epitopes from SAPs

From the antigenic *C. dubliniensis* SAPs, B-cell, T-helper (T_h_) cell, and T-cytotoxic cell (T_c_) epitopes were predicted using the IEDB B-cell epitope prediction tool, and the NetMHCII 2.3 and NETMHCpan 4.0 web servers, respectively [27,28,29]. To predict B-cell epitopes, SAP sequences were input in plain format and the Bepipred linear epitope prediction method was utilized [29]. However, there are limitations with the Bepipred-based B-cell epitope prediction method as it can only predict linear B-cell epitopes while most of the antigenic B-cell epitopes are discontinuous [30,31]. For the NetMHCII 2.3 and NetMHCpan 4.0 web servers, SAPs were used in FASTA format and 9-mer epitopes were predicted using default parameters. The human leukocyte antigen (HLA) alleles used while predicting the T_h_ and T_c_ epitopes are listed in Supplementary Tables S1 and S2. T_h_ and T_c_ epitopes that were determined as strong binders were chosen in the NetMHCII 2.3 and NetMHCpan 4.0 web servers for further analyses. 

#### 2.2.2. Selection of the Best Epitopes against *C. dubliniensis*

The epitopes’ antigenicity, allergen potential, toxicity, and capacity to activate interleukin-2 (IL2), interleukin-4 (IL4), and IFN-γ (IFN-γ) were predicted using the Vaxijen 2.0, AllergenFP, ToxinPred, IL2Pred, IL4Pred, and IFNepitope computational tools, respectively [26,32,33,34,35,36]. While using these web servers, the default parameters were kept. In the Vaxijen 2.0 web server, fungi were chosen as target organisms. 

#### 2.2.3. Analysis of the Population Coverage of the Selected Epitopes

In order to determine population coverage analysis for the selected T_h_ and T_c_ epitopes for the vaccine candidate design, the IEDB population coverage analysis tool (available at http://tools.iedb.org/population/, accessed on 30 December 2022) was used. Notably, the population coverage analysis for the final B-cell epitopes could not be determined due to the lack of web servers/software that could predict the B-cell epitope population coverage. In the IEDB population coverage analysis tool, the default values of the “number of epitopes” and “query by” box were chosen. “World” was chosen for “select area(s) and/or population(s)”. Under “select calculation option”, the Class I and II combined option was selected.

#### 2.2.4. Designing the Final Vaccine Construct

B-cell and T-cell epitopes that were predicted to activate IFN-γ, IL2, IL4, and to be antigenic, non-allergenic, and non-toxic were selected for final vaccine design. *Salmonella dublin* flagellin protein, RS09 (APPHALS), and Pan HLA DR-binding epitope (PADRE) were used as adjuvants. The adjuvants and epitopes (both B-cell and T-cell) were joined using “GGS” linkers. The antigenicity, allergenic potential and physiochemical properties, such as stability, isoelectric point, and extinction co-efficient of the final *C. dubliniensis* vaccine candidate, were determined using Vaxijen 2.0, AllergenFP, and ExPASyProtParam, respectively [26,34,37]. We have previously used a similar approach for vaccine design for a canine circovirus, dengue virus, and monkeypox virus, and pathogenic fungi such as *Candida auris* and *Candida tropicalis* [24,38,39,40,41].

### 2.3. Molecular Modeling, Docking, and Molecular Dynamics Simulations Study

The 3D structure prediction of the fungal multi-epitope vaccine (MEV) and of human TLR5 (Uniprot ID: D1CS82) was performed using AlphaFold v.2 [42,43]. Quality of the predicted tertiary structures of MEV and TLR5 was checked with the ProSA webserver. For docking fungal MEV and TLR-5, the HADDOCK server [44] was used, using default settings. The docking protocol similar to that in our previous studies has been performed [38,39,40]. In particular, we used an information-driven docking methodology, based on the information about specific interacting residues, to drive the docking simulations. A recent study has clearly demonstrated the potential binding regions for flagellin (inserted in the fungal MEV construct) and human TLR5 were LQRVRELAVQ and EILDISRNQL [45]. Thus, during the docking experiments, these peptides were used to define active residues in running docking simulations. The top-ranked cluster containing the lowest HADDOCK score was selected as a final structure for subsequent analyses.

Molecular dynamics simulations (MD) were carried out with GROMACS 2022 [46]. The fungal *Candida dubliniensis* multi-epitope vaccine construct complexed with TLR5 was mapped to a coarse-grained model using cgconv from sirah suite tools (Machado et al., 2019). The coarse-grained complex of MEV-TLR5 was placed in a cubic box and solvated using a coarse-grained WatFour model (WT4) [47], a water model with coarse-grained ions NaW (Na^+^), and ClW (Cl^−^). The SIRAH force field [48] was used to obtain the parameters of the proteins and solvent model. Charges were neutralized by adding Na and Cl ions using 0.15 M near a physiological concentration to achieve a bulk ionic strength. The simulation box contained 1750 Na^+^ ions, 1743 Cl^−^, and 56,112 WT4 water molecules, respectively. The total number of atoms in the system was 64,725. The simulation protocol consisted of the following steps: (1) solvent and side chains relaxation by 5000 steps of energy minimization, imposing positional restraints of 1000 kJ mol^−1^ nm^−2^ on backbone beads corresponding to the nitrogen and carboxylic oxygen (named GN and GO, respectively); (2) full system relaxation by 5000 steps of unrestrained energy minimization; (3) solvent equilibration by 5 ns of MD in the NVT ensemble at 310 K, imposing positional restraints of 1000 kJ mol^−1^ nm^−2^ on the whole protein; (4) protein relaxation by 25 ns of MD in the NVT ensemble at 310 K, imposing positional restraints of 100 kJ mol^−1^ nm^−2^ on GN and GO beads; (5) production simulation in the NPT ensemble at 310 K and 1 bar. Non-bonded interactions were treated with a 1.2 nm cutoff and PME for long-range electrostatics. A time step of 20 fs was used in MD simulations. Snapshots were recorded every 100 ps for analysis. For the simulation, PME and neighbor searching were computed every 10 integration steps, setting a Fourier spacing of 0.2 nm and considering a Verlet cutoff scheme of 1.4 nm. Automatic tuning of these options was not allowed when the Verlet–buffer–drift flag was set to −1. Solvent and solute were coupled separately to V-rescale [49] thermostats with coupling times of 2 ps. The system’s pressure was controlled by a Parrinello–Rahman barostat [50] with a coupling time of 8 ps. The backmapping to convert the coarse-grained frames to all atom models was performed using sirah_vmdtk.tcl of the sirah tools suite and VMD [51]. For visualization and for creating molecular graphics images, the Chimera USFC software [52] was used.

### 2.4. In silico Immunosimulation of the C. dubliniensis Vaccine Candidate

In silico immune simulation was performed by the C-IMMSIM online tool using default settings (except time step) for investigating the immune response profile elicited by the *C. dubliniensis* vaccine candidate in the recipients [53]. It has been advised that the least duration between two consecutive vaccine dose administrations could be four weeks; but in some cases, a minimum duration of 8 weeks to 6 months could be used [54,55]. Therefore, the immune response profile elicited for the *C. dubliniensis* vaccine candidate was determined by administering three vaccine doses every four weeks. The time steps of 1, 84 (equivalent to 4 weeks), and 168 (equivalent to 8 weeks) were used.

## 3. Results

### 3.1. C. dubliniensis SAPs Sequence Retrieval and Analysis

Eight proteins are classified as SAPs in *C. dubliniensis*, SAP1, SAP2, SAP3, SAP6, SAP7, SAP8, SAP9, and SAP10, as it misses orthologs for SAP4 and SAP5 of *Candida albicans* [14,17]. The accession number and antigenicity of the *C. dubliniensis* SAP proteins are listed in Table 1. Vaxijen 2.0 predicted all the *C. dubliniensis* SAPs except SAP7 as antigenic. Only those *C. dubliniensis* SAPs that were predicted as antigenic were selected for further analyses.

### 3.2. Epitope Prediction from SAPs for Vaccine Candidate Design

From the seven antigenic *C. dubliniensis* SAPs, 437 strong binding T_h_ cell epitopes were predicted (Table S1). Similarly, 213 strong binding T_c_ epitopes were predicted from the antigenic *C. dubliniensis* SAPs (Table S2). Altogether, 39 B-cell epitopes were predicted from the *C. dubliniensis* SAPs using the IEDB B-cell epitope prediction tool (Table S3).

### 3.3. Best Epitopes Prediction for Vaccine Candidate Design against C. dubliniensis and Population Coverage Analysis

Finally, eight epitopes (2 B-cell, 3 T_h_, and 3 T_c_) were selected for vaccine design as they were predicted as antigenic, non-allergenic, non-toxic, and were able to activate the production of interferon-γ, IL-2, and IL4 (Table 2). The epitopes that were selected for final vaccine design could provide 66.41% of global population coverage.

### 3.4. Design of Final C. dubliniensis Vaccine Construct

**Figure 1:**
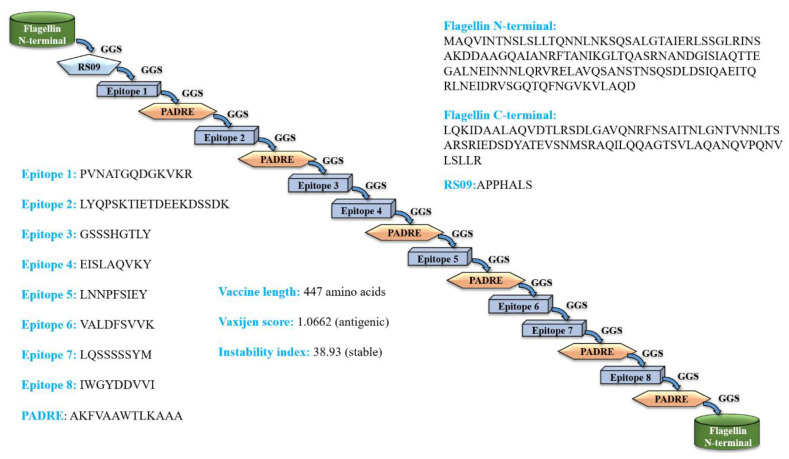
Scheme of the predicted full-fledged constructed fungal multi-epitope vaccine (MEV) construct. (PADRE = Pan HLA DR-binding epitope).

The eight epitopes and adjuvants were joined by GGS linkers to design a stable, antigenic and non-allergenic *C. dubliniensis* vaccine construct that contains 447 amino acids. The amino acid sequence of the vaccine construct is presented below; see Figure 1. The epitopes chosen for vaccine design are in bold font. Further, the physiochemical properties of the final vaccine construct, such as isoelectric point, number of atoms, theoretical pI, aliphatic index, etc., are provided in Table S4.

### 3.5. Modeling and Docking of TLR5 Fungal–MEV Construct

**Figure 2:**
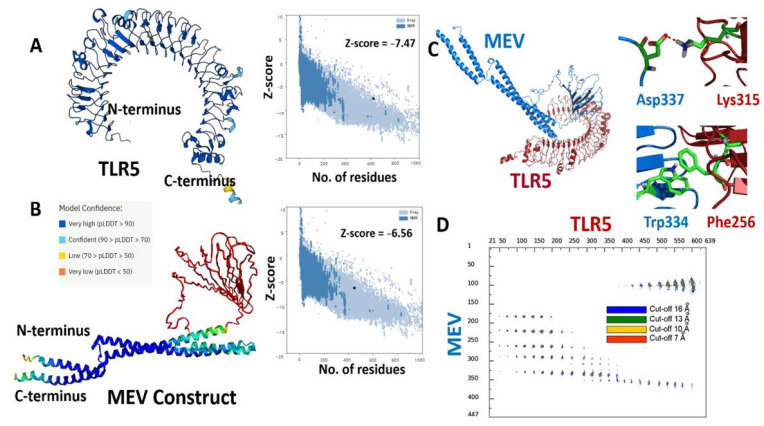
(**A**) Cartoon representations of modeled 3D structures for (**A**) human TLR5; and (**B**) the fungal MEV construct with respective Z-scores calculated by ProSA. (**C**) Cartoon representation of the predicted complex between TLR5 (red) and fungal MEV (blue). (**D**) Distance contact map indicating the residues in contact in the predicted MEV–TLR5 complex at different distance thresholds, along with a stick representation of two representative molecular interactions between them.

Alphafoldv2.0 program was used to predict the three-dimensional (3D) structures for both the fungal MEV construct and the immunogenic human TLR5 receptor [42,43]. The TLR5 structure was predicted with a very high expected accuracy, with confidence scores (predicted local distance difference test, pLDDT values) > 90 for most of the residues (see Figure 2A). We particularly targeted the ectodomain residues (amino acids from 22–639) of the topological domain, which are primarily involved in the interaction with the extracellular signal. The fungal MEV Alphafold model also resulted in high pLDDT values >90 for the N- and C-terminal regions, where the flagellin protein was inserted in the MEV construct. However, low pLDDT scores with values <50 were predicted for the regions where PADRE/linkers and epitope peptide were present (amino acids from 142–303); see Figure 2B. Further, the quality of predicted 3D structures for both immunogenic TLR5 and fungal MEV constructs was confirmed and validated by calculated Z-scores of −7.47 and −6.56 for the TLR5 and the fungal MEV constructs, respectively, from the ProSA web server [56]. The modeled structures of the TLR5 and fungal MEV constructs were subjected to molecular docking using the HADDOCK 2.4 web server [44]. The molecular docking between the fungal–MEV constructs and the TLR5 receptor is shown in Figure 2C, where two instances of different interactions are also illustrated. Further, the distance range maps calculated using the COCOMAPS tool [57,58] clearly demonstrated a number of contacts stabilizing the interface between the docked MEV and TLR5 molecules; see Figure 2D.

### 3.6. Stability of Vaccine Construct Complexed with TLR5 Receptor

**Figure 3:**
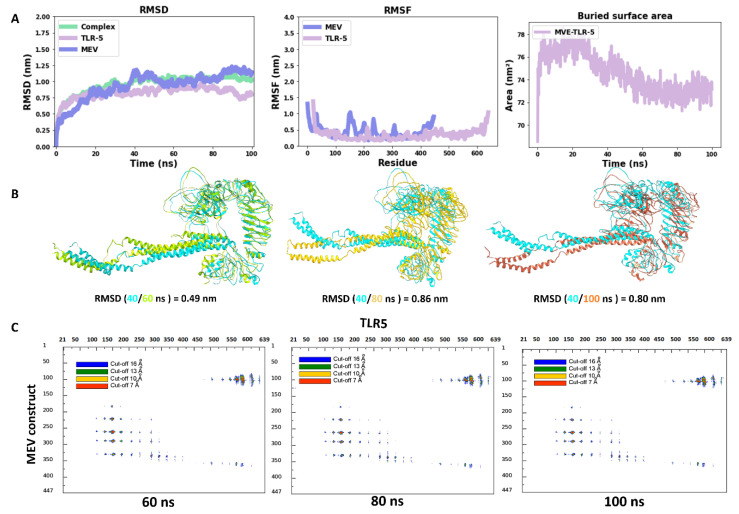
(**A**) Time evolution of the backbone RMSD of the TLR5, MEV constructs and their complex during MD simulations, backbone RMSF plots, buried surface area; (**B**) superimposition of snapshots of TLR5 and MEV constructs with their respective RMSD values; (**C**) contact maps showing inter-molecular contacts where the dots at the crossover of two amino acids have been colored in red, yellow, green, and blue if any pair of atoms between two amino acids was closer than 7, 10, 13, or 16 Å.

**Figure 4:**
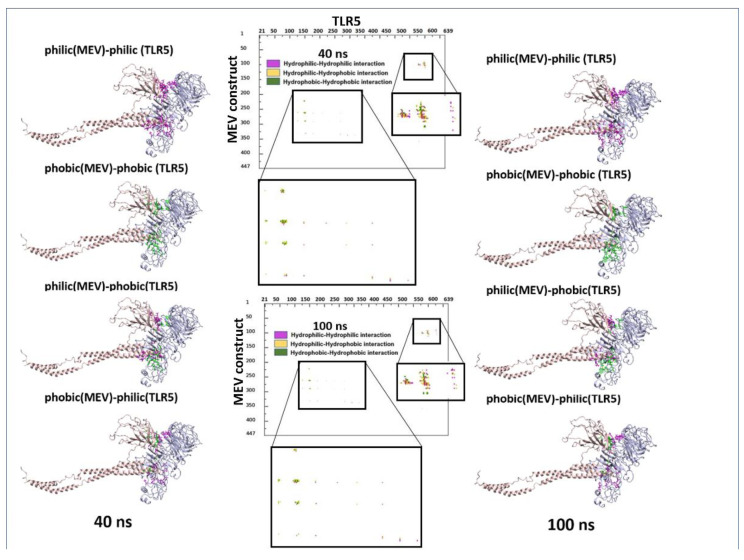
Property contact map of TLR5 and fungal MEV constructs for snapshots at 40 ns and 100 ns. Structures with the highlighted hydrophobic (phobic) and hydrophilic (philic) residues present at the interfaces are shown in green and magenta colors, respectively.

Molecular dynamics has shown to be an advantageous method for studying the stability of biological systems [59]. In this work, we have used the GROMACS software to carry out MD simulations for assessing the stability of the multi-epitope vaccine complexed with TLR5. A single 100 ns-long simulation of the complex was carried out. All the calculated parameters for the simulation are reported in Figure 3A. The root mean square deviation (RMSD) of the backbone from the first position, a handy benchmark for indicating the complex stability or possible conformational drift, was calculated. The high RMSD of the complex was observed, with an average value of 0.95 ± 0.14 nm (Figure 3A). The highly flexible multi-epitope vaccine construct possessed a high RMSD due to the terminal N- and C- flagellin molecule, with an average RMSD of 0.93 ± 0.21 nm (Figure 3A). The TLR5 receptor seemed very stable, with an average RMSD value of 0.82 ± 0.09 nm (Figure 3A). Looking at Figure 3A (RMSD plot), it seems evident that the complex started to attain stability after 40 ns of simulation and remained stable until the 100 ns simulation time. Next, we separately plotted the root mean square fluctuation (RMSF) for the TLR5 and the vaccine construct. It is clear that certain residues, especially in the peripheral regions of MEV, are associated with an elevated RMSF values with an average value of 0.43 ± 0.19 nm. Moreover, a high RMSF values is observed for regions where the epitope/adjuvants were inserted (155–334 amino acids) (Figure 3A). Besides, the limited flexibility of TLR5 is clearly reflected by low RMSF average values of 0.33 ± 0.14 nm (Figure 3A). It is further illustrated from Figure 3A that the buried surface area at the interface of the vaccine construct and TLR5 remained stable throughout the simulation time, with an average of 74.5 ± 1.7 nm^2^. The above analyses demonstrated clearly the stability of interface interactions between the TLR5 and the vaccine construct. The superimposition was performed to study further the structural stability of the overall complex, resulting in a good overlap between selected structures extracted at different time steps. The high RMSD of structures indicated in Figure 3B was clearly due to the regions, corresponding to the N- and C-terminal flagellin molecules, that seem very flexible in the vaccine construct. Nevertheless, the interaction pattern of residues in contact between the vaccine construct and the TLR5 remained similar. Furthermore, an interface analysis of selected snapshots was performed comparing TLR5 and the docked vaccine construct using the COCOMAPS tool [57,58] (Figure 3C). Contact maps were computed, where the dots at the crossover of two residues belonging to the vaccine construct and TLR5 were colored in red, yellow, green, and blue if any pair of their atoms were closer than 7, 10, 13, or 16 Å. Figure 3C shows that the interface remained stable for the selected snapshots in terms of inter-residue contacts, notwithstanding the observed flexibility in the N- and C-terminal regions of the vaccine construct, which was clearly not interacting with TLR5. Further, the property contact map was calculated (see Figure 4), where the contacts were colored according to physicochemical nature of involved residues for selected snapshots at 40 ns and 100 ns. The structures with the highlighted hydrophobic (phobic) and hydrophilic (philic) residues present at the interfaces are shown in green and magenta color, respectively. It is clear from the analysis that the hydrophobic–hydrophilic interactions contributed most to the interface, with a number of contacts corresponding to 40/100 ns of simulation time of 135/122; they are then followed in number by the hydrophilic–hydrophilic, 64/59, and by the hydrophobic–hydrophobic, 34/26, contacts.

### 3.7. In Silico Immunosimulation of the C. dubliniensis Vaccine Candidate

**Figure 5:**
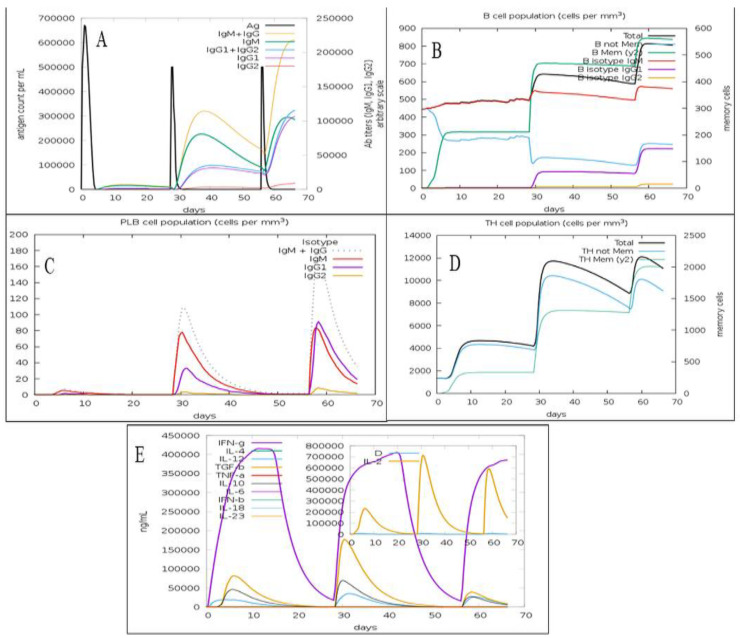
Immune profile of the fungal MEV candidate showing (**A**) antigen count and antibody titer with Ig subclass; (**B**) B-cell population; (**C**) plasma B-cell population; (**D**) T_h_ population per state; (**E**) cytokines and interleukins population.

The predicted immune response profile of the *C. dubliniensis* vaccine candidate is presented in Figure 5. The second and third doses of the vaccine increased the concentration of IgM, IgG1, IgG2, IgG1 + IgG2, and IgM + IgG antibodies in comparison to the first dose (Figure 5A). The consecutive vaccine administrations also increased the total B-cell population and B-memory cell population, suggesting the elicitation of a strong secondary immune response (Figure 5B). The plasma B lymphocyte population after first dose of vaccine was observed to be very low but after the second and third vaccination, their populations (IgG1, IgM, and IgM+IgG) significantly increased (Figure 5C). The plasma B lymphocyte population plays an important role in adaptive immunity [60]. The total T_h-_cell population along with T_h_ memory cells increased after second dose in comparison to first dose (Figure 5D). However, the total T_h-_cell population remained similar following the second and third injection of the vaccine candidate. Furthermore, the administration of *C. dubliniensis* vaccine candidate has potential to activate the production of cytokines such as IFN-gamma, transforming growth factor-beta (TGF-β), interleukin-10 (IL10), and interlukin-12 (IL12) (Figure 5E). Overall, the second and third doses of the vaccine candidate that were administered after 4 weeks and 8 weeks increased the antibody titer, B-cell population, plasma B-lymphocyte population, and T_h-_cell population in comparison to the first dose.

## 4. Discussion

Although *C. dubliniensis* is less virulent than *C. albicans*, the public health concern of *C. dubliniensis* cannot be overlooked due to its increased resistance to antifungal drugs and also its correlation with several health complications such as meningitis, endocarditis, spondylodiscitis, and oral and respiratory candidiasis, in immunocompromised patients [1,6,11]. Thus, it is urgently required to seek novel and effective drugs, immunotherapy, and vaccines to prevent and cure *C. dubliniensis* infections. Plant-derived DNA topoisomerase inhibitors such as curcumin, etoposide, and camptothecin have shown strong antifungal activity against *C. dubliniensis* [61]. Similarly, a potential immunotherapy that used complement receptor 3-related protein (CR3-RP) antibody has shown anti-biofilm activity against *C. dubliniensis* [62]. However, a vaccine targeting *C. dubliniensis* has not been reported yet. Thus, we conducted a study using an in silico approach towards finding a novel multi-epitope vaccine candidate against *C. dubliniensis*. Recently, the usage of immunoinformatics for developing vaccine candidates has significantly increased, since this in silico-based approach is time- and cost-saving in formulating novel vaccine candidates [41,42,43,44,45,63]. Interestingly, similar in silico strategies have been used to design vaccine candidates against other *Candida* species including *C. albicans*, *C. auris,* and *C. tropicalis* [24,41,64]. Tarang et al. (2020) targeted ALS2, ALS3, ALS4, HYR1, FAV2, HWP1, EAP1, and SAP2 proteins of *C. albicans* to identify B-cell and T-cell epitopes; they identified 18 epitopes for preparing a novel vaccine construct [64]. Further, Akhtar et al. (2021) developed a novel vaccine candidate against the highly virulent and rapidly spreading pathogenic fungi *C. auris* by targeting its ALS3 protein [41]. Akhtar et al. (2020) predicted 3 B-cell epitopes, 3 T_c_-cell epitopes and 2 T_h_r-cell epitopes to predict a vaccine for *C. auris* [41]. A multi-epitope vaccine candidate against *C. tropicalis* was developed by identifying IFN-γ activating, non-allergenic, antigenic, and non-toxic epitopes from the protein SAP2 of *C. tropicalis* [24].

Herein, the SAP proteins of *C. dubliniensis* were investigated while using various computational tools to predict epitopes that could be potentially used for vaccine candidate development. In various pathogenic *Candida* species, SAPs act as the virulence factor and help in hypha formation, nutrient acquisition, adherence, and invasion of host cells [16,18]. Previously, SAP-based vaccine candidates have provided immunization against infections from *C. tropicalis* and *C. albicans* [19,21,23]. Eight epitopes (see Table 2) were selected for final vaccine construct design. These eight epitopes were predicted as non-allergenic, antigenic, non-toxic, and showed potential to elicit iIL2, IL4, and IFN-γ. These epitopes were then linked by GGS linkers with PADRE (AKFVAAWTLKAAA), RS09 and *S. dublin* flagellin adjuvants to design the final *C. dubliniensis* vaccine candidate. This is the first study that reports a *C. dubliniensis* vaccine construct. The final *C. dubliniensis* vaccine candidate has been predicted to be stable, antigenic, and non-allergenic. The immune simulation analysis depicts that the second and third doses of the vaccine candidate that were administered after 4 weeks and 8 weeks increased the total antibody titer, B-cell population, plasma B-lymphocyte population, and T_h_-cell population compared to the first dose. Finally, molecular docking and molecular dynamics simulations confirmed stable interactions between the vaccine candidate and human TLR5. The computational analyses performed in this study show the immunogenic potential of the *C. dubliniensis* vaccine candidate. However, further in vivo studies are needed to establish the safety and efficacy of the epitopes and the proposed vaccine candidate. In future, studies similar to that of Kaushik et al. (2022) can be performed where the immunogenic epitopes predicted in this study can be synthesized and their ability to generate protective antibodies in animal models can be assessed [38]. Furthermore, the proposed vaccine candidate can be cloned and expressed as recombinant protein. The recombinant vaccine candidate can be used to immunize mice models infected with candidiasis and the ability of the vaccine candidate to elicit immune response and protect the mice from *Candida* infection can be determined by following a methodology similar to that of Shukla et al. (2022) [25].

## 5. Conclusions

Using an immunoinformatics approach, a novel vaccine candidate has been developed against *C. dubliniensis*, which is predicted to be stable, non-allergenic, and antigenic. The population coverage analysis showed that the vaccine candidate could provide immunization for two-thirds of the global population. The predicted vaccine candidate is antigenic and non-allergenic in nature and interacts strongly with human TLR5. Furthermore, immune simulation predicted that the vaccine candidate could elicit robust immunization in recipients.

## Figures and Tables

**Table 1:** *C. dubliniensis* SAP proteins and their antigenicity.

Protein	Accession Number	Vaxijen Score/Antigenicity
SAP1	XP_002421073.1	0.6952 /Antigen
SAP2	XP_002422286.1	0.7204/Antigen
SAP3	XP_002419429.1	0.7361/Antigen
SAP6	XP_002421072.1	0.6642/Antigen
SAP7	XP_002417130.1	0.3687/Non-antigen
SAP8	XP_002419185.1	0.6518/Antigen
SAP9	XP_002419306.1	0.8219/Antigen
SAP10	XP_002420070.1	0.5786/Antigen

**Table 2:** Final epitopes selected for vaccine design and their properties.

Epitope Type	Protein ID	Peptide	Binding Affinity (nM)	Vaxijen Score	Antigen/Non-Antigen	Allergenicity	Toxicity	IL-2 Inducer	IL-4 Inducer	IFNepitope
B-cell	XP_002421073.1	PVNATGQDGKVKR	NA	1.7534	Antigen	Non-allergen	Non-toxin	Inducer	Inducer	Yes
B-cell	XP_002419306.1	LYQPSKTIETDEEKDSSDK	NA	0.5936	Antigen	Non-allergen	Non-toxin	Inducer	Inducer	Yes
T_c_-cell	XP_002421073.1	GSSSHGTLY	144.8	0.9695	Antigen	Non-allergen	Non-toxin	Inducer	Inducer	Yes
	XP_002421073.1	EISLAQVKY	572.7	0.8786	Antigen	Non-allergen	Non-toxin	Inducer	Inducer	Yes
	XP_002419429.1	LNNPFSIEY	1028.1	2.8077	Antigen	Non-allergen	Non-toxin	Inducer	Inducer	Yes
T_h_-cell	XP_002422286.1	VALDFSVVK	5122.8	1.4652	Antigen	Non-allergen	Non-toxin	Inducer	Inducer	Yes
	XP_002419306.1	LQSSSSSYM	207.3	0.8152	Antigen	Non-allergen	Non-toxin	Inducer	Inducer	Yes
	XP_002419306.1	IWGYDDVVI	2881.8	0.5754	Antigen	Non-allergen	Non-toxin	Inducer	Inducer	Yes

## Data Availability

The data presented in this study are available in the article and the Supplementary Materials.

## Supplementary Materials

The following supporting information can be downloaded at: https://www.mdpi.com/article/10.3390/vaccines11020364/s1, Table S1: Protein IDs with corresponding predicted T_h_-cell epitopes with their corresponding calculated parameters; Table S2: Protein ids with corresponding predicted T_c_-cell epitopes with their corresponding calculated parameters; Table S3: Protein ids with corresponding predicted B-cell epitopes with their corresponding calculated parameters; Table S4: Physiochemical properties of vaccine candidate.
