# Supplementary material for: Secreted Aspartyl Proteinases Targeted Multi-Epitope Vaccine Design for Candida dubliniensis Using Immunoinformatics
Source: Vaccines (Basel). 2023 Feb 5;11(2):364. doi: 10.3390/vaccines11020364 (PMC9964391; doi:10.3390/vaccines11020364)
Supplement: Supplementary file 1 [file vaccines-11-00364-s001.zip › vaccines-2168424-supplementary.pdf]

Secreted Aspartyl Proteinases **Targeted Multiepitope Vaccine Design for Candida dubliniensis using Immunoinformatics**

Nahid Akhtar[1], Jorge Samuel Leon Magdaleno[2],Suryakant Ranjan[3], Atif Khurshid Wani[3], Ravneet Kaur Grewal[1], Romina Oliva[4], Abdul Rajjak Shaikh[1,*], Luigi Cavallo[2,*], Mohit Chawla[2,*]

[1]Department of Research and Innovation, STEMskills Research and Education Lab Private Limited, Faridabad 121002, Haryana, India, [2]King Abdullah University of Science and Technology (KAUST), Physical Sciences and Engineering Division, Kaust Catalysis Center, Thuwal 23955-6900, Saudi Arabia.[3]School of Bio-Engineering and Bio-Sciences, Lovely Professional University, Punjab, India [4]Department of Sciences and Technologies, University Parthenope of Naples, Centro Direzionale Isola C4, I-80143, Naples, Italy.

Email: mohitchawla.bt@gmail.com; Mohit.chawla@kaust.edu.sa; luigi.cavllo@kaust.edu.sa; ab_rajjak@yahoo.co.in

Table S1: Protein ids with corresponding predicted T_h cell epitopes with their corresponding calculated parameters

Protein ID	Allele	Peptide	Binding Affinity (nM)	Vaxijen score	Antigen/Non-antigen	Allergenicity	Toxicity	IL-2 inducers	IL-4 inducer	IFN epitope
XP_002421073.1	DRB1_0101	DNFLRSAYI	458.8	-0.7148	Non-antigen					
		FEVIKTPVN	884.8	0.5796	Antigen	Non-allergen	Non-Toxin	non-inducer		
		FNFANNAKI	1288.5	1.9797	Antigen	Allergen				
	DRB1_0301	LLVDATPAK	1992.1	0.8572	Antigen	Allergen				
		ILGDNFLRS	4556.4	0.3729	Non-antigen					
		PVTSDRELR	5130.2	2.1556	Antigen	Non-Allergen	Non-Toxin	non-inducer		
		VTLDFEVIK	5558.1	1.7315	Antigen	Non-Allergen	Non-Toxin	non-inducer		

		LVDATPAKR	6639.7	1.2091	Antigen	Allergen				
		ALLVDATPA	7538.3	0.1407	Non-antigen					
		NILGDNFLR	7598.4	0.7548	Antigen	Allergen				
		VTSDRELRI	7896.3	2.8970	Antigen	Non-Allergen	Non-Toxin	non-inducer		
		VFADVTKTS	8033.9	-1.3433	Non-antigen					
		VLLDSGTTI	8439.5	-1.2202	Non-antigen					
		ITLNSIKAA	9810.5	0.3230	Non-antigen					
		VTLNNEVVS	9821.0	0.3759	Non-antigen					
	DRB1_0401	LYLNSPNAA	1495.4	1.8544	Antigen	Non-Allergen	Non-Toxin	Inducer	Non-inducer	
		YTSASNIAA	1977.6	1.6527	Antigen	Allergen				
		YAADITVGS	2706.6	2.2518	Antigen	Allergen				
		LLLGINDAN	2799.3	0.3488	Non-antigen					
		FALLVDATP	3262.3	0.6164	Antigen	Non-Allergen	Non-Toxin	Inducer	Non-inducer	
		LYKDTVGFG	3779.0	0.7262	Non-antigen	Allergen				
		LLVDATPAK	4181.5	0.8572	Non-antigen	Allergen				
		YSLYLNSPN	4884.6	2.7280	Antigen	Non-Allergen	Non-Toxin	Inducer	Non-inducer	
		KYTSASNIA	4986.4	1.5271	Antigen	Non-Allergen	Non-Toxin	Inducer	Inducer	No
		LRITLNSIK	6742.4	0.3099	Non-antigen					
		FIALAFALL	6931.1	-1.2598	Non-antigen					
		ILGDNFLRS	7196.5	0.3729	Non-antigen					
		LKNIFIALA	7603.3	-0.2144	Non-antigen					
	DRB1_0701	FNFANNAKI	21.7	1.9797	Antigen	Allergen				
		VKYTSASNI	55.5	1.3080	Antigen	Allergen				
		LFYTNGQPY	155.5	-2.0996	Non-antigen					
	DRB1_0801	FEVIKTPVN	738.5	0.5796	Antigen	Non-Allergen	Non-Toxin	non-inducer		
	DRB1_0901	FNFANNAKI	204.4	1.9797	Antigen	Allergen				
		QRLGNPFYI	772.0	1.8625	Antigen	Non-Allergen	Non-Toxin	Inducer	Non-inducer	
		FCKGKGLYT	815.2	0.7052	Antigen	Allergen				
		AKNAYSLYL	1039.3	2.3423	Antigen	Non-Allergen	Non-Toxin	Inducer	Non-inducer	
		ISLAQVKYT	1153.0	0.3063	Non-antigen					
	DRB1_1001	YSLYLNSPN	387.7	2.7280	Antigen	Non-Allergen	Non-Toxin	Inducer	Non-inducer	
		FIALAFALL	608.1	-1.2598	Non-antigen					

		FEVIKTPVN	739.8	0.5796	Antigen	Non-Allergen	Non-Toxin	non-inducer		
		QRLGNPFYI	1280.9	1.8625	Antigen	Non-Allergen	Non-Toxin	Inducer	Non-inducer	
		FNFANNAKI	1299.3	1.9797	Antigen	Allergen				
		YTSASNIAA	1461.9	1.6527	Antigen	Allergen				
		YFQQDVAQG	1476.4	0.6098	Antigen	Allergen				
	DRB1_1201	EISLAQVKY	457.3	0.8786	Antigen	Non-allergen	Non-Toxin	Inducer	Inducer	Yes
		VIAKNAYSL	824.4	1.1626	Antigen	Allergen				
		IAKNAYSLY	1015.5	1.2062	Antigen	Allergen				
	DRB1_1301	ITVGSNRQK	36.6	0.8698	Antigen	Non-Allergen	Non-Toxin	Inducer	Inducer	No
	DRB1_1501	KNIFIALAF	4846.0	-0.1472	Non-antigen					
	DRB1_1602	FEVIKTPVN	2987.8	0.5796	Antigen	Non-Allergen	Non-Toxin	non-inducer		
		FCKGKGLYT	4577.0	0.7052	Antigen	Allergen				
		FYIGYGDGS	4867.7	0.3848	Non-antigen					
		YTSASNIAA	4929.0	1.6527	Antigen	Allergen				
		YSLYLNSPN	5127.4	2.7280	Antigen	Non-Allergen	Non-Toxin	Inducer	Non-inducer	
		FIALAFALL	5316.4	-1.2598	Non-antigen	Allergen				
		SLYLNSPNA	5481.5	1.9820	Antigen	Allergen				
XP_002422286.1	DRB1_0101	FSVVKTPKA	231.6	0.2835	Non-antigen					
		DNFLRSAYI	458.8	-0.7148	Non-antigen					
		FVALDFSVV	740.5	-0.0489	Non-antigen					
		ITYLQQDLA	1297.4	0.2152	Non-antigen					
		FNFSKNAKI	1508.6	3.0085	Antigen	Allergen				
	DRB1_0301	LQTDDGQTY	4519.0	2.0692	Antigen	Allergen				
		ILGDNFLRS	4556.4	0.3729	Non-antigen					
		VALDFSVVK	5122.8	1.4652	Antigen	Non-Allergen	Non-Toxin	Inducer	Inducer	Yes
		VLADISSTS	5845.1	-0.0736	Non-antigen					
		NILGDNFLR	7598.4	0.7548	Antigen	Allergen				
		LLVDATPTT	7860.5	0.4365	Non-antigen					
		VLLDSGTTI	8439.5	-1.2202	Non-antigen					
		QLLFDVNDA	9217.7	1.4332	Antigen	Allergen				
		ALLVDATPT	9947.4	-0.1166	Non-antigen					

		INTDNVDVL	10250.5	2.2948	Antigen	Allergen				
	DRB1_0401	YLVDCNVSG	1337.0	2.0307	Antigen	Allergen				
		LYLNSPNAA	1495.4	1.8544	Antigen	Non-Allergen	Non-Toxin	Inducer	Non-inducer	
		YTSESSISA	1538.5	1.7220	Antigen	Allergen				
		FYLVDCNVS	1750.4	1.9914	Antigen	Allergen				
		YAADITVGS	2706.6	2.2518	Antigen	Allergen				
		LLFDVNDAN	3575.9	1.3427	Antigen	Non-allergen	Non-toxin	Non-inducer		
		LLVDATPTT	3709.0	0.4365	Non-antigen					
		LYKDTVGFG	3779.0	0.7262	Antigen	Allergen				
		YSLYLNSPN	4884.6	2.7280	Antigen	Non-Allergen	Non-Toxin	Inducer	Non-inducer	
		VLADISSTS	4916.3	-0.0736	Non-antigen					
		FSVVKTPKA	6098.9	0.2835	Non-antigen					
		ILGDNFLRS	7196.5	0.3729	Non-antigen					
		FVALDFSVV	7449.0	-0.0489	Non-antigen					
		FIALAIALL	7542.6	-1.6002	Non-antigen					
		LKNIFIALA	7603.3	-0.2144	Non-antigen					
	DRB1_0701	FNFSKNAKI	7.7	3.0085	Antigen	Allergen				
		FSVVKTPKA	50.3	0.2835	Non-antigen					
	DRB1_0801	FSVVKTPKA	440.9	0.2835	Non-antigen					
	DRB1_0901	FNFSKNAKI	283.2	3.0085	Antigen	Allergen				
		FIALAIALL	414.6	-1.6002	Non-antigen					
		LRISLGSVE	635.3	1.0764	Antigen	Non-Allergen	Non-Toxin	Inducer	Inducer	No
		AKNAYSLYL	1039.3	2.3423	Antigen	Non-Allergen	Non-Toxin	Inducer	Non-inducer	
		ISLAQVKYT	1153.0	0.3063	Non-antigen					
		FSVVKTPKA	1213.6	0.2835	Non-antigen					
		VVKAFNGEL	1220.2	2.9469	Antigen	Non-Allergen	Non-Toxin	non-inducer		
	DRB1_1001	FIALAIALL	157.6	-1.6002	Non-antigen					
		YSLYLNSPN	387.7	2.7280	Antigen	Non-Allergen	Non-Toxin	Inducer	Non-inducer	
		FVALDFSVV	1122.8	-0.0489	Non-antigen					
		VKAFNGELT	1504.7	3.7836	Antigen	Non-Allergen	Non-Toxin	non-inducer		
	DRB1_1201	EISLAQVKY	457.3	0.8786	Antigen	Non-Allergen	Non-Toxin	Inducer	Inducer	Yes
		VIAKNAYSL	824.4	0.1626	Antigen	Allergen				

		IAKNAYSLY	1015.5	1.2062	Antigen	Allergen				
	DRB1_1501	KNIFIALAI	2714.9	-0.5022	Non-antigen					
	DRB1_1602	FSVVKTPKA	1515.4	0.2835	Non-antigen					
		FIALAIALL	4376.1	-1.6002	Non-antigen					
		YSLYLNSPN	5127.4	2.7280	Antigen	Non-Allergen	Non-Toxin	Inducer	Non-inducer	
		SLYLNSPNA	5481.5	1.9820	Antigen	Allergen				
XP_002419429.1	DRB1_0101	DNFLRSAYI	458.8	-0.7148	Non-antigen					
		FVALNFDVI	1590.6	0.4788	Non-antigen					
	DRB1_0301	ILGDNFLRS	4556.4	0.3729	Non-antigen					
		NILGDNFLR	7598.4	0.7548	Antigen	Allergen				
		FAFDKNAKI	8253.1	2.8615	Antigen	Allergen				
		VLLDSGTTI	8439.5	-1.2202	Non-antigen					
		TLLNEQVSY	9748.3	-1.5464	Non-antigen					
	DRB1_0401	FYLVDCNLS	868.4	1.9516	Antigen	Allergen				
		YLVDCNLSG	1204.3	2.0007	Antigen	Allergen				
		YASDITVGS	1814.8	2.2550	Antigen	Allergen				
		YTTASNIAA	2350.3	1.5991	Antigen	Non-Allergen	Non-Toxin	Inducer	Non-inducer	
		WYKDTIGFG	3835.1	0.3524	Non-antigen					
		KYTTASNIA	4884.4	1.5933	Antigen	Non-Allergen	Non-Toxin	Inducer	Inducer	No
		IKTHKNVTG	4937.6	3.0294	Antigen	Non-Allergen	Non-Toxin	non-inducer		
		LLNEQVSYA	5820.9	-0.7659	Non-antigen					
		LITLPTTSN	5966.1	0.3321	Non-antigen					
		FADVTSTSV	6294.5	-0.0970	Non-antigen					
		LKNIFITLA	6396.3	-0.3461	Non-antigen					
		ILGDNFLRS	7196.5	0.3729	Non-antigen					
		LLVDAIPTT	7278.3	-0.2254	Non-antigen					
		VIKTHKNVT	7327.9	0.5833	Non-antigen	Non-allergen	Non-toxin	Non-inducer		
	DRB1_0701	VKYTTASNI	25.6	1.3190	Antigen	Allergen				
		CNLSGSVEF	60.4	1.0650	Antigen	Allergen				
	DRB1_0801	FDVIKTHKN	831.9	0.2574	Non-antigen					
	DRB1_0901	FITLAIALL	678.9	-1.2241	Non-antigen					
		CNLSGSVEF	695.7	1.0650	Antigen	Allergen				

		AKNAYSLYL	1039.3	2.3423	Antigen	Non-Allergen	Non-Toxin	Inducer	Non-inducer	
		ISLAQVKYT	1153.0	0.3063	Non-antigen					
	DRB1_1001	FITLAIALL	564.4	-1.2241	Non-antigen					
		LITLPTTSN	1282.9	0.3321	Non-antigen					
		QVKYTTASN	1630.7	1.0572	Antigen	Allergen				
		NFDVIKTHK	3965.0	1.3243	Antigen	Non-Allergen	Non-Toxin	non-inducer		
	DRB1_1201	EISLAQVKY	457.3	0.8786	Antigen	Non-Allergen	Non-Toxin	Inducer	Inducer	Yes
		IIAKNAYSL	501.5	1.1412	Antigen	Allergen				
		IAKNAYSLY	1015.5	1.2062	Antigen	Allergen				
	DRB1_1301	LRIHLNTVT	60.9	1.5984	Antigen	Allergen				
	DRB1_1501	KNIFITLAI	3395.9	-0.5021	Non-antigen					
	DRB1_1602	YLNSRQSAS	5275.4	1.4337	Antigen	Allergen				
XP_002421072.1	DRB1_0101	IIYALGAQV	395.7	0.1022	Non-antigen					
		DNFMRSAYI	477.9	-0.7700	Non-antigen					
		FNVMKTPVD	672.5	1.0203	Antigen	Non-Allergen	Non-Toxin	Inducer	Non-inducer	
		SVNVLGQNV	965.2	2.3186	Antigen	Allergen				
		SVALKSVNV	1107.4	0.0491	Non-antigen					
		FIALDFNVM	1166.2	1.9407	Antigen	Allergen				
		FLQNILSVL	1207.4	-0.0848	Non-antigen					
	DRB1_0301	IALDFNVMK	2415.8	3.4038	Antigen	Allergen				
		LLIDAAPVK	4137.8	0.9224	Antigen	Allergen				
		ILGDNFMRS	4195.7	0.5691	Antigen	Non-allergen	Non-toxin	Inducer	Inducer	No
		DLDDNKISM	4206.5	1.6916	Antigen	Allergen				
		VDLDNEIIT	5090.7	1.8800	Antigen	Allergen				
		ITSDMRLSV	6843.4	2.5932	Antigen	Allergen				
		IVYDLDDNK	7039.2	2.9649	Antigen	Allergen				
		NILGDNFMR	7320.7	1.0089	Antigen	Allergen				
		VLLDSGTTI	8439.5	-1.2202	Non-antigen					
		YDLDDNKIS	9023.9	2.1088	Antigen	Non-Allergen	Non-Toxin	Inducer	Inducer	No
		LIDAAPVKR	9734.0	0.9044	Antigen	Non-Allergen	Non-Toxin	Inducer	Inducer	No
		IFADVWSTS	10378.7	0.4001	Non-antigen					
		PITSDMRLS	10461.4	2.0009	Antigen	Allergen				

	DRB1_0401	YAADITLGS	2072.3	2.1100	Antigen	Allergen				
		FIALDFNVM	2400.9	1.9407	Antigen	Allergen				
		FQFDKNLKI	2961.6	4.2801	Antigen	Non-Allergen	Non-Toxin	Inducer	Inducer	No
		IALDFNVMK	3647.6	3.4038	Antigen	Allergen				
		LFLDIDHTG	4865.9	0.2691	Non-antigen					
		LYQDTVGLG	6313.0	1.1822	Antigen	Allergen				
		ILGDNFMRS	6720.5	0.5691	Antigen	Non-allergen	Non-toxin	Inducer	Inducer	No
		VRASESNIL	7194.8	2.1763	Antigen	Allergen				
		FADVWSTSA	7307.8	0.6188	Antigen	Allergen				
	DRB1_0701	MRLSVALKS	41.0	0.0769	Non-antigen					
		FQPSIARNI	42.1	1.5179	Antigen	Allergen				
		IIYALGAQV	54.3	0.1022	Non-antigen					
		FQFDKNLKI	88.9	4.2801	Antigen	Non-Allergen	Non-Toxin	Inducer	Inducer	No
		VRASESNIL	147.2	2.1763	Antigen	Allergen				
		LYYTNGEPY	265.7	-1.1743	Non-antigen					
	DRB1_0801	FNVMKTPVD	271.4	1.0203	Antigen	Non-Allergen	Non-Toxin	Inducer	Non-inducer	
	DRB1_0901	IIYALGAQV	51.8	0.1022	Non-antigen					
		ISYFQPSIA	145.7	0.2193	Non-antigen					
		VKRSPGFIA	682.8	0.2880	Non-antigen					
		MRLSVALKS	796.5	0.0769	Non-antigen					
		ILSVLAVAL	1198.3	-0.8110	Non-antigen					
		IQYADGSYA	1200.7	0.3229	Non-antigen					
	DRB1_1001	YFQPSIARN	496.3	0.1048	Non-antigen					
		FIALDFNVM	712.5	1.9407	Antigen	Allergen				
		FIAPLYYTN	772.4	0.6606	Antigen	Allergen				
		FLQNILSVL	863.5	-0.0848	Non-antigen					
		ISYFQPSIA	940.3	0.2193	Non-antigen					
		YSLFLNSPE	1124.3	1.9003	Antigen	Allergen				
		FNVMKTPVD	1359.7	1.0203	Antigen	Non-Allergen	Non-Toxin	Inducer	Non-inducer	
		KISMAPVKY	1405.8	0.9678	Antigen	Non-Allergen	Non-Toxin	Inducer	Inducer	No
	DRB1_1101	MRLSVALKS	1760.8	0.0769	Non-antigen					
		LPISMKKQG	3423.7	-0.3080	Non-antigen					
	DRB1_1201	IISKNAYSL	654.3	1.1884	Antigen	Allergen				
		VRAKRSPLF	744.5	0.4167	Non-antigen					

		KISMAPVKY	1067.6	0.9678	Antigen	Non-Allergen	Non-Toxin	Inducer	Inducer	No
		SVALKSVNV	1109.6	0.0491	Non-antigen					
		IIYALGAQV	1515.8	0.1022	Non-antigen					
	DRB1_1301	MRLSVALKS	27.6	0.0769	Non-antigen					
		FMRSAYIVY	42.4	-0.8316	Non-antigen					
	DRB1_1501	IIYALGAQV	324.3	0.1022	Non-antigen					
	DRB1_1602	FQFDKNLKI	3821.4	4.2801	Antigen	Non-Allergen	Non-Toxin	Inducer	Inducer	No
		FLQNILSVL	4387.9	-0.0848	Non-antigen					
		FIALDFNVM	4783.5	1.9407	Antigen	Allergen				
		SLFLNSPEA	5103.2	1.2572	Antigen	Non-Allergen	Non-Toxin	non-inducer		
XP_002419185.1	DRB1_0101	FNFAKGVTI	291.9	1.2688	Antigen	Allergen				
		FALLAQGLA	563.6	-1.7523	Non-antigen					
		ITFTKNVLV	1281.0	1.3861	Antigen	Allergen				
		TGSLTALPI	1362.1	1.4861	Antigen	Allergen				
		SLYLNSASA	1494.0	0.3165	Non-antigen					
	DRB1_0301	ILGDNFLRR	1157.5	0.6629	Antigen	Non-allergen	Inducer	Non-inducer		
		VSLDFTVTR	2195.4	2.1928	Antigen	Non-Allergen	Non-Toxin	Inducer	Inducer	No
		VLQDGNSCY	5165.9	-1.3037	Non-antigen					
		TILGDNFLR	5725.3	0.4585	Antigen	Allergen				
	DRB1_0401	YTSSCNLSG	2195.6	1.2123	Antigen	Allergen				
		FAKGVTISV	2456.5	1.8396	Antigen	Allergen				
		YSLYLNSAS	2524.3	1.0637	Antigen	Non-Allergen	Non-Toxin	Inducer	Non-inducer	
		YLNSASASS	3184.2	0.5536	Antigen	Allergen				
		LYTSSCNLS	3188.2	1.4223	Antigen	Allergen				
		YTTSSSIST	3414.3	2.0743	Antigen	Allergen				
		LYLNSASAS	4141.2	0.4779	Non-antigen					
		YAATITVGS	5119.1	1.1851	Antigen	Allergen				
		FVLQDGNSC	5989.2	-0.5260	Non-antigen					
		VQLSTINIA	6273.4	0.7202	Antigen	Non-Allergen	Non-Toxin	Inducer	Non-inducer	
		FADVTTTSI	6574.8	-0.5297	Non-antigen					
		YYQLYTSSC	6970.7	0.9649	Antigen	Non-Allergen	Non-Toxin	Inducer	Non-inducer	
	DRB1_0701	FNFAKGVTI	8.3	1.2688	Antigen	Allergen				
		ITFTKNVLV	21.4	1.3861	Antigen	Allergen				
		VKYTTSSSI	26.5	1.2955	Antigen	Allergen				

		KGVTISVPL	118.3	2.9703	Antigen	Allergen				
		FSQTIADKL	135.2	-0.9443	Non-antigen					
		LPITSSNEL	247.0	1.5083	Antigen	Allergen				
		AEKVVSLDF	312.4	2.4086	Antigen	Allergen				
	DRB1_0801	FTVTRKPFN	302.2	0.8944	Antigen	Allergen				
		FLRRAYAVY	691.7	0.6191	Antigen	Non-Allergen	Non-Toxin	Inducer	Non-inducer	
		FALLAQGLA	768.8	-1.7523	Non-antigen					
		FNFAKGVTI	771.1	1.2688	Antigen	Allergen				
		DFTVTRKPF	874.6	2.7000	Antigen	Non-Allergen	Non-Toxin	Inducer	Inducer	No
	DRB1_0901	FNFAKGVTI	166.9	1.2688	Antigen	Allergen				
		ALLAQGLAI	696.7	-0.6422	Non-antigen					
		AFALLAQGL	774.5	-1.9549	Non-antigen					
		VKYTTSSSI	776.1	1.2955	Antigen	Allergen				
		LRRAYAVYD	913.4	0.6369	Antigen	Non-Allergen	Non-Toxin	Inducer	Non-inducer	
		LGKAFSIQY	1035.5	4.5208	Antigen	Non-Allergen	Non-Toxin	Inducer	Inducer	No
		ISLAQVKYT	1153.0	0.3063	Non-antigen					
	DRB1_1001	YSLYLNSAS	659.1	1.0637	Antigen	Non-Allergen	Non-Toxin	Inducer	Non-inducer	
		YFSQTIADK	752.7	-1.8456	Non-antigen					
		YYQLYTSSC	793.3	0.9649	Antigen	Non-Allergen	Non-toxin	Inducer	Non-inducer	
		YAATITVGS	853.2	1.1851	Antigen	Allergen				
		YLNSASASS	917.6	0.5536	Antigen	Allergen				
		AFALLAQGL	1653.3	-1.9549	Non-antigen					
	DRB1_1101	FALLAQGLA	2987.8	-1.7523	Non-antigen					
		YFGVSRDSA	3613.0	1.7871	Antigen	Non-Allergen	Non-Toxin	Inducer	Inducer	No
	DRB1_1201	TISLAQVKY	170.2	0.4755	Non-antigen					
		IINKNAYSL	692.0	0.3997	Non-antigen					
		INKNAYSLY	1359.6	0.8450	Antigen	Allergen				
		LWVIDSAAV	1928.1	0.4765	Non-antigen					
		AFALLAQGL	1985.2	-1.9549	Non-antigen					
	DRB1_1301	FLRRAYAVY	30.2	0.6191	Antigen	Non-Allergen	Non-Toxin	Inducer	Non-inducer	
		IITFTKNVL	61.4	0.0064	Non-antigen					
	DRB1_1501	IITFTKNVL	2197.7	0.0064	Non-antigen					
		IVFNFAKGV	3669.4	-0.4898	Non-antigen					

		LRRAYAVYD	4314.5	0.6369	Antigen	Non-Allergen	Non-Toxin	Inducer	Non-inducer	
		ITFTKNVLV	4788.9	1.3861	Antigen	Allergen				
	DRB1_1602	YSLYLNSAS	2031.2	1.0637	Antigen	Non-Allergen	Non-Toxin	Inducer	Non-inducer	
		YYQLYTSSC	3211.6	0.9649	Antigen	Non-Allergen	Non-Toxin	Inducer	Non-inducer	
		LWVIDSAAV	3328.6	0.4765	Non-antigen					
		YLNSASASS	3950.7	0.5536	Antigen	Allergen				
		LYLNSASAS	4387.3	0.4779	Non-antigen					
		SLYLNSASA	5247.2	0.3165	Non-antigen					
XP_002419306.1	DRB1_0101	DNILRSAYI	343.4	-1.2703	Non-antigen					
		LVLQAGRST	456.9	-0.0958	Non-antigen					
		YSRSVGAYI	587.8	2.3929	Antigen	Allergen				
		FYMATLKIG	768.0	-1.0905	Non-antigen					
		LSFAIANET	792.7	0.3221	Non-antigen					
		GIIAKSLYS	820.0	-1.2680	Non-antigen					
		GIGLPGLEV	884.2	1.3368	Antigen	Non-Allergen	Non-Toxin	Non-inducer		
		YELLLSLLV	943.2	-0.2083	Non-antigen					
		LLSLVATAL	1219.1	-1.6212	Non-antigen					
		LSLVATALA	1342.9	-0.7118	Non-antigen					
		FGHGTGVKL	1454.1	5.1289	Antigen	Non-Allergen	Non-Toxin	Inducer	Inducer	No
		SYMLFGDNI	1493.7	2.4644	Antigen	Allergen	Non-Toxin	non-inducer		
		TFYMATLKI	1598.5	-0.8843	Non-antigen					
	DRB1_0301	MLFGDNILR	1041.7	-0.4587	Non-antigen					
		LFGDNILRS	1775.0	-0.6090	Non-antigen					
		FKIDFEVRR	2388.4	4.0706	Antigen	Allergen				
		VKRDGSLDM	3799.3	-0.2475	Non-antigen					
		VKLNERELL	4188.7	0.3780	Non-antigen					
		MSHDLKCVS	4665.0	0.7753	Antigen	Allergen				
		MRLNSVALL	5169.1	-0.3058	Non-antigen					
		LKADGIIAK	5340.5	-0.4177	Non-antigen					
		VVISNVTVK	5885.6	-0.8969	Non-antigen					
		LLQKRKNLY	8005.7	0.1840	Non-antigen					
		WGYDDVVIS	10291.9	0.0007	Non-antigen					
		VMSHDLKCV	10365.8	0.9801	Antigen	Non-allergen	Non-toxin	Non-inducer		

		MTLTNKQTF	10485.3	-0.3736	Non-antigen				
	DRB1_0401	LFGDNILRS	1456.4	-0.6090	Non-antigen				
		VMLQSSSSS	1778.8	0.1853	Non-antigen				
		YIVNCNLAD	2884.9	0.8797	Antigen	Allergen			
		LKADGIIAK	3418.9	-0.4177	Non-antigen				
		VKMMRTYSQ	4395.0	1.7438	Antigen	Non-Allergen	Non-Toxin	non-inducer		
		YVFSDTLES	4546.6	-0.0415	Non-antigen				
		MLQSSSSSY	4839.3	0.2578	Non-antigen				
		LYSLYLNSA	5024.7	0.5498	Antigen	Non-Allergen	Non-Toxin	Inducer	Inducer	No
		AYIVNCNLA	5723.0	1.6761	Antigen	Allergen			
		GVMLQSSSS	6075.4	-0.2978	Non-antigen				
		WGYDDVVIS	6466.8	0.0007	Non-antigen				
		YLNSADAKA	7016.9	1.9136	Antigen	Allergen			
		FAIANETSS	7090.9	0.8806	Antigen	Allergen			
	DRB1_0701	YSRSVGAYI	11.4	2.3929	Antigen	Allergen			
		LATTAPFKI	63.0	0.5613	Antigen	Allergen			
		IEVIGASGI	69.8	-0.1658	Non-antigen				
		LQSSSSSYM	207.3	0.8152	Antigen	Non-Allergen	Non-Toxin	Inducer	Inducer	Yes
		TFYMATLK	219.7	-1.3896	Non-antigen				
		LQAGRSTCI	245.2	2.8295	Antigen	Allergen			
		FGHGTGVKL	255.4	5.1289	Antigen	Non-Allergen	Non-Toxin	Inducer	Inducer	No
		LSFAIANET	273.8	0.3221	Non-antigen				
		LSLVATALA	312.7	-0.7118	Non-antigen				
	DRB1_0801	FYMATLKIG	192.6	-1.0905	Non-antigen				
		LVTVKMMRT	350.7	0.1990	Non-antigen				
		TFYMATLKI	373.3	-1.3896	Non-antigen				
		LWVMSHDLK	738.1	3.2205	Antigen	Allergen			
	DRB1_0901	YSRSVGAYI	49.2	2.3929	Antigen	Allergen			
		LQSSSSSYM	202.1	0.8152	Antigen	Non-Allergen	Non-Toxin	Inducer	Inducer	Yes
		MLQSSSSSY	376.1	0.2578	Non-antigen				
		FGHGTGVKL	597.8	5.1289	Antigen	Non-Allergen	Non-Toxin	Inducer	Inducer	No
		LLSLVATAL	611.6	-1.6212	Non-antigen				

		MRLNSVALL	703.4	-0.3058	Non-antigen					
		LATTAPFKI	847.1	0.5613	Antigen	Allergen				
		AYSTFSPFV	912.3	0.7045	Antigen	Allergen				
		TRHSAGSII	1013.2	2.4237	Antigen	Allergen				
		YSTFSPFVG	1208.7	1.4934	Antigen	Allergen				
	DRB1_1001	YMATLKIGS	300.1	0.3131	Non-antigen					
		VKMMRTYSQ	614.1	1.7438	Antigen	Non-Allergen	Non-Toxin	non-inducer		
		YEVSLAQVS	662.2	0.4008	Non-antigen					
		YSRSVGAYI	870.5	2.3929	Antigen	Allergen				
		TVKMMRTYS	934.6	1.2009	Antigen	Allergen				
		LLSLVATAL	1139.5	-1.6212	Non-antigen					
		YELLLSLLV	1183.3	-0.2083	Non-antigen					
		YSLYLNSAD	1476.6	1.7845	Antigen	Allergen				
	DRB1_1101	YMATLKIGS	215.7	0.3131	Non-antigen					
		FYMATLKIG	869.7	-1.0905	Non-antigen					
		TVKMMRTYS	2508.8	1.2009	Antigen	Allergen				
		RELLQKRKN	3469.0	0.4633	Non-antigen					
		YQNLPLKLK	4201.2	-0.6437	Non-antigen					
	DRB1_1201	VTVKMMRTY	270.1	1.2038	Antigen	Non-Allergen	Non-Toxin	non-inducer		
		IAKSLYSLY	520.9	0.6837	Antigen	Allergen				
		EVSLAQVSY	782.2	0.0927	Non-antigen					
		IIAKSLYSL	1314.5	-0.5713	Non-antigen					
		LLQKRKNLY	1594.5	0.1840	Non-antigen					
		LLSLVATAL	2158.6	-1.6212	Non-antigen					
	DRB1_1301	LVTVKMMRT	22.4	0.1990	Non-antigen					
		LLQKRKNLY	23.5	0.1840	Non-antigen					
		VRIQVPVSK	31.5	1.8396	Antigen	Allergen				
		LVLQAGRST	32.4	-0.0958	Non-antigen					
		VTVKMMRTY	38.1	1.2038	Antigen	Non-Allergen	Non-Toxin	non-inducer		
		MRLNSVALL	39.9	-0.3058	Non-antigen					
		ILRSAYIVY	50.8	-1.0241	Non-antigen					
		MRTYSQISY	62.3	-0.6069	Non-antigen					
		LLSLVATAL	65.2	-1.6212	Non-antigen					
	DRB1_1501	YSRSVGAYI	950.3	2.3929	Antigen	Allergen				

		AKSLYSLYL	1936.3	1.2197	Antigen	Allergen				
		IFGFGSVYS	2261.3	-0.6350	Non-antigen					
		IWGYDDVVI	2881.8	0.5754	Antigen	Non-allergen	Non-toxin	Inducer	Inducer	Yes
		LSLLVAYCV	3024.6	1.4798	Antigen	Non-Allergen	Non-Toxin	Inducer	Non-inducer	
		YMLFGDNIL	3067.9	1.0484	Antigen	Allergen				
		LLSLLVAYC	3359.2	0.8941	Antigen	Allergen				
		SVALLSLVA	3479.0	-0.5179	Non-antigen					
		LLSLVATAL	4882.9	-1.6212	Non-antigen					
		YELLLSLLV	4968.3	-0.2083	Non-antigen					
	DRB1_1602	YMLFGDNIL	2347.9	1.0484	Antigen	Allergen				
		YELLLSLLV	2429.2	-0.2083	Non-antigen					
		YMATLKIGS	3247.8	0.3131	Antigen	Allergen				
		LLSLVATAL	4710.8	-1.6212	Non-antigen					
		IFGFGSVYS	5342.7	-0.6350	Non-antigen					
		AKSLYSLYL	5436.0	1.2197	Antigen	Allergen				
		GVMLQSSSS	5440.0	-0.2978	Non-antigen					
XP_002420070.1	DRB1_0101	YLAIMSNSV	159.0	0.0106	Non-antigen					
		LAIMSNSVI	281.7	-0.9463	Non-antigen					
		WIDALGRSL	1055.4	0.3362	Non-antigen					
		LKIGIANRS	1125.1	-1.2872	Non-antigen					
	DRB1_0301	LFGDDILRR	238.2	-0.2876	Non-antigen					
		ILFGDDILR	686.6	-0.2307	Non-antigen					
		VLLDTGSTF	2548.2	-2.5278	Non-antigen					
		IKLDFNIVS	2687.0	1.9926	Antigen	Allergen				
		LVYDLQDMT	3253.1	0.8536	Antigen	Allergen				
		IFNDIDQEY	3435.2	-0.0513	Non-antigen					
		VMSNDAICY	3763.8	-0.8864	Non-antigen					
		PLINDKILY	3821.1	-1.4843	Non-antigen					
		YLVYDLQDM	9571.3	0.4453	Non-antigen					
		IFPDEWIDA	10094.6	-0.0672	Non-antigen					
	DRB1_0401	IKLDFNIVS	931.6	1.9926	Antigen	Allergen				
		YLAIMSNSV	2161.8	0.0106	Non-antigen					
		VYLNSSNST	3543.1	1.2584	Antigen	Allergen				

		LFGDDILR R	4677.4	-0.2876	Non-antigen					
		LKIGIANRS	5241.8	-1.2872	Non-antigen					
		YSVYLNSS N	6203.4	1.5311	Antigen	Allergen				
		YLNSSNST T	7227.6	1.9050	Antigen	Non-Allergen	Non-Toxin	Inducer	Non-inducer	
	DRB1_070 1	YLAIMSNS V	45.8	0.0106	Non-antigen					
		ILYTTELEI	114.8	0.8142	Antigen	Allergen				
	DRB1_080 1	LYLASVVK C	267.0	-1.4903	Non-antigen					
		LLYLASVV K	852.6	-2.2039	Non-antigen					
	DRB1_090 1	FFGFSIGNA	960.1	0.0797	Non-antigen					
		MTISVAPV A	973.5	0.7395	Antigen	Allergen				
		WIDALGRS L	1189.7	0.3362	Non-antigen					
	DRB1_100 1	YLAIMSNS V	121.7	0.0106	Non-antigen					
		YSVYLNSS N	470.4	1.5311	Antigen	Allergen				
		FLLYLASV V	1024.2	-1.6177	Non-antigen					
		FFGFSIGNA	1131.2	0.0797	Non-antigen					
	DRB1_110 1	YLAIMSNS V	2710.3	0.0106	Non-antigen					
		YLASVVKC S	4377.9	-1.1056	Non-antigen					
	DRB1_120 1	INKIAYSVY	375.9	-0.6434	Non-antigen					
		LINKIAYSV	764.2	-1.5125	Non-antigen					
		VMSNDAIC Y	1003.0	-0.8864	Non-antigen					
		ILRRIYLVY	1710.6	-0.2414	Non-antigen					
		PLINDKILY	1863.2	-1.4843	Non-antigen					
		YLAIMSNS V	1951.2	0.0106	Non-antigen					
	DRB1_130 1	ILRRIYLVY	26.1	-0.2414	Non-antigen					
		LKIGIANRS	36.5	-1.2872	Non-antigen					
		LLYLASVV K	63.1	-2.2039	Non-antigen					
	DRB1_150 1	NKIAYSVY L	1527.3	0.5472	Antigen	Allergen				
		YLAIMSNS V	1965.9	0.0106	Non-antigen					
		VWGYDSV QF	2310.5	-0.4405	Non-antigen					
		LAIMSNSVI	2317.5	-0.9463	Non-antigen					
		FLLYLASV V	3003.0	-1.6177	Non-antigen					
		RRIYLVYDL	4647.7	1.9460	Antigen	Allergen				

	DRB1_1602	YLAIMSNSV	684.5	0.0106	Non-antigen					
		FLLYLASVV	1788.5	-1.6177	Non-antigen					
		LWVMSNDAI	3399.7	1.3804	Antigen	Allergen				
		FFGFSIGNA	4042.9	0.0797	Non-antigen					
		YSVYLNSSN	4482.6	1.5311	Antigen	Allergen				
		CYLAIMSNS	5422.4	0.6931	Antigen	Allergen				

Table S2: Protein ids with corresponding predicted T_c cell epitopes with their corresponding calculated parameters

Protein ID	Allele	Peptide	Binding Affinity (nM)	Vaxijen score	Antigen/ Non-antigen	Allergenicity	Toxicity	IL-2 inducer	IL-4 inducer	IFNepitope
XP_002421073.1	HLA-A*01:01	GSSSHGTLY	144.8	0.9695	Antigen	Non-allergen	Non-Toxin	Inducer	Inducer	Yes
		KTNEAAGDY	549.7	1.2788	Antigen	Allergen				
		ASEFTASLF	686.8	0.2726	Non-antigen					
	HLA-A*02:01	FLKNIFIAL	26.5	-0.1943	Non-antigen					
	HLA-A*03:01	SSSHGTLYK	41.9	0.4811	Non-antigen					
	HLA-A*26:01	EISLAQVKY	572.7	0.8786	Antigen	Non-allergen	Non-Toxin	Inducer	Inducer	Yes
		EVVSYAADI	639.4	1.9098	Antigen	Allergen				
		DTVGFGGAS	1327.2	1.3930	Antigen	Allergen				
	HLA-B*07:02	SPNAATGQI	24.8	1.8632	Antigen	Allergen				
		TPAKRSAGF	32.7	0.2214	Non-antigen					
		VPVTSDREL	180.1	2.2722	Antigen	Non-allergen	Non-Toxin		Non-Inducer	
	HLA-B*08:01	FLKNIFIAL	19.4	-0.1943	Non-antigen					
		DGKVKRQAI	50.0	0.0835	Non-antigen					
		SNRQKFNVV	52.6	0.7537	Antigen	Non-allergen	Non-Toxin		Inducer	No
	HLA-B*27:05	KRQAIPVTL	49.1	0.1663	Non-antigen					
		KRSAGFVTL	56.2	0.3515	Non-antigen					
		QRLGNPFYI	118.9	1.8625	Antigen	Non-allergen	Non-Toxin		Non-Inducer	
		LRITLNSIK	128.5	0.3099	Non-antigen	Allergen				
	HLA-B*39:01	NRQKFNVVV	117.3	-0.0053	Non-antigen					
		KRSAGFVTL	179.8	0.3515	Non-antigen					
		KQDGNGNSL	304.8	2.8548	Antigen	Allergen				
		QRLGNPFYI	332.6	1.8625	Antigen	Non-allergen	Non-Toxin		Non-Inducer	
		KRQAIPVTL	396.0	0.1663	Non-antigen					
	HLA-B*58:01	RSAYIVYDL	36.9	-0.2506	Non-antigen					
XP_002422286.1	HLA-A*01:01	GSSSQGTLY	161.2	0.9782	Antigen	Non-allergen	Non-Toxin		Inducer	No
		KTNEAGGDY	554.5	1.5254	Antigen	Non-allergen	Non-Toxin		Non-Inducer	
		NTDNVDVLL	627.5	1.6703	Antigen	Allergen				
	HLA-A*02:01	FLKNIFIAL	26.5	-0.1943	Non-antigen					
		FIALAIALL	34.7	-1.6002	Non-antigen					
	HLA-A*03:01	SSSQGTLYK	81.7	0.1643	Non-antigen					
	HLA-A*24:02	TYSKCQLLF	15.6	-0.4281	Non-antigen					
		KYSGSLITL	113.2	1.8834	Antigen	Non-allergen	Non-Toxin		Inducer	No
		TYSDQTADF	213.0	-0.4834	Non-antigen					
	HLA-A*26:01	EISLAQVKY	572.7	0.8786	Antigen	Non-allergen	Non-Toxin	Inducer	Inducer	Yes
		DTVGFGGAS	1327.2	1.3930	Antigen	Allergen				

	HLA-B*07:02	SPNAATGQI	24.8	1.8632	Antigen	Allergen				
		LPVTSNTEL	49.8	1.6314	Antigen	Non-allergen	Non-Toxin		Inducer	No
	HLA-B*08:01	FLKNIFIAL	19.4	- 0.1943	Non-antigen					
	HLA-B*27:05	KRSAGFVAL	46.2	- 0.0738	Non-antigen					
		KRQAIPVTL	49.1	0.1663	Non-antigen					
	HLA-B*39:01	KRSAGFVAL	97.9	- 0.0738	Non-antigen					
		KRQAIPVTL	396.0	0.1663	Non-antigen					
	HLA-B*58:01	RSAYIVYDL	36.9	- 0.2506	Non-antigen					
		VSGDVVFNF	58.4	0.1217	Non-antigen					
XP_002419429.1	HLA-A*01:01	ASEFTAPLY	27.4	1.4721	Antigen	Non-allergen	Non-Toxin		Inducer	No
		YDANGNLFY	342.8	0.5718	Antigen	Allergen				
		GTTSQGTWY	702.6	- 0.2215	Non-antigen					
		LNNPFSIEY	1028.1	2.8077	Antigen	Non-allergen	Non-Toxin	Inducer	Inducer	Yes
	HLA-A*02:01	FLKNIFITL	26.7	- 0.2901	Non-antigen					
		LLNEQVSYA	27.1	- 0.7659	Non-antigen					
		LLVDAIPTT	37.6	- 0.2254	Non-antigen					
	HLA-A*24:02	VYDQCQLLF	75.5	- 0.9237	Non-antigen					
		TWYKDTIGF	96.7	- 0.4828	Non-antigen					
		KYSGSLITL	113.2	1.8834	Antigen	Non-allergen	Non-Toxin		Inducer	No
		TYDANGNLF	116.7	1.8277	Antigen	Allergen				
		YYDNVPVTL	284.9	- 0.0769	Non-antigen					
	HLA-A*26:01	ETYDANGNL	156.0	1.7976	Antigen	Non-allergen	Non-Toxin		Inducer	No
		TTASNIAAL	174.0	1.7343	Antigen	Non-allergen	Non-Toxin		Non-Inducer	
		EISLAQVKY	572.7	0.8786	Antigen	Non-allergen	Non-Toxin	Inducer	Inducer	Yes
	HLA-B*07:02	LPTTSNSEL	43.8	0.7389	Antigen	Non-allergen	Non-Toxin		Inducer	No
	HLA-B*08:01	FLKNIFITL	31.6	- 0.2901	Non-antigen					
		ELRIHLNTV	57.8	2.0948	Antigen	Allergen				
		LAQVKYTTA	147.2	0.4669	Non-antigen					
		NVKRQTVPV	151.9	0.5811	Antigen	Allergen				
	HLA-B*27:05	KRQTVPVTL	81.4	0.5626	Antigen	Allergen				
	HLA-B*39:01	YYDNVPVTL	228.2	- 0.0769	Non-antigen					
	HLA-B*58:01	LSGSVEFAF	21.3	1.5882	Antigen	Allergen				
		RSAYIVYDL	36.9	- 0.2506	Non-antigen					
XP_002421072.1	HLA-A*01:01	ASEFIAPLY	25.2	1.3635	Antigen	Non-allergen	Non-Toxin		Inducer	No
		DLDNEIITY	343.6	1.1393	Antigen	Non-allergen	Non-Toxin		Inducer	No

		GSYARGNLY	702.3	-1.7592	Non-antigen				
		FADVWSTSA	1027.9	0.6188	Antigen	Allergen			
	HLA-A*02:01	FLQNILSVL	12.2	-0.0848	Non-antigen				
		ALLIDAAPV	21.8	0.0127	Non-antigen				
		FQFDKNLKI	35.0	4.2801	Antigen	Non-allergen	Non-Toxin		Inducer	No
	HLA-A*03:01	GILGIGFQK	94.2	-0.2299	Non-antigen				
	HLA-A*26:01	DTVGLGGAF	10.3	2.7130	Antigen	Allergen			
		FIALDFNVM	1015.6	1.9407	Antigen	Allergen			
		NIQYADGSY	1123.4	-0.2216	Non-antigen				
	HLA-B*07:02	TVRAKRSPL	29.2	1.1786	Antigen	Non-allergen	Non-Toxin		Non-Inducer	
		QPSIARNII	42.0	0.9904	Antigen	Non-allergen	Non-Toxin		Inducer	No
		APVKRSPGF	52.3	0.8451	Antigen	Non-allergen	Non-Toxin		Inducer	No
		SPEASTGQI	137.8	1.9382	Antigen	Allergen			
		IARNIIYAL	167.4	-0.8004	Non-antigen				
	HLA-B*08:01	TVRAKRSPL	20.9	1.1786	Antigen	Non-allergen	Non-Toxin		Non-Inducer	
		IARNIIYAL	131.4	-0.8004	Non-antigen				
		FLQNILSVL	184.1	-0.0848	Non-antigen				
	HLA-B*27:05	KRSPGFIAL	67.9	-0.5262	Non-antigen				
		KRSPLFLDI	82.4	-2.1045	Non-antigen				
	HLA-B*39:01	KRSPGFIAL	131.8	-0.5262	Non-antigen	Non-allergen	Non-Toxin		Non-Inducer	No
		VRASESNIL	215.7	2.1763	Antigen	Allergen			
		FQFDKNLKI	246.4	4.2801	Antigen	Non-allergen	Non-Toxin		Inducer	No
		SKNAYSLFL	316.7	1.5126	Antigen	Allergen			
		FLQNILSVL	492.9	-0.0848	Non-antigen				
	HLA-B*58:01	RSAYIVYDL	36.9	-0.2506	Non-antigen				
		TSGTVDFQF	74.3	0.3215	Non-antigen				
		ISVPASEFI	81.4	1.9174	Antigen	Allergen			
		YALGAQVHF	82.2	3.6766	Antigen	Allergen			
XP_002419185.1	HLA-A*01:01	GSSSQGTVY	863.0	1.3521	Antigen	Allergen			
	HLA-A*02:01	VLVALAFAL	21.4	-0.0040	Non-antigen				
	HLA-A*03:01	KLAAAVGAK	13.3	1.3284	Antigen	Allergen			
		MVSIITFTK	37.6	-0.0476	Non-antigen				
		ATITVGSNK	56.0	1.6427	Antigen	Allergen			
		SLDFTVTRK	131.3	2.1115	Antigen	Non-allergen	Non-Toxin		Inducer	No
		SSSQGTVYK	138.8	0.5699	Antigen	Allergen			
	HLA-A*24:02	TYPGQSPTF	36.2	2.0790	Antigen	Allergen			

	HLA-A*26:01	FTKNVLVAL	757.5	0.9724	Antigen	Non-allergen	Non-Toxin		Non-Inducer	
		FLRRAYAVY	1400.2	0.6191	Antigen	Non-allergen	Non-Toxin		Non-Inducer	
	HLA-B*07:02	HPAQKRGTV	8.5	0.4227	Non-antigen					
		LPITSSNEL	41.2	1.5083	Antigen	Allergen				
	HLA-B*08:01	NFLRRAYAV	78.0	0.8450	Antigen	Allergen				
		ELRVQLSTI	155.8	1.7528	Antigen	Non-allergen	Non-Toxin	Non-inducer	Inducer	Yes
		HPAQKRGTV	203.8	0.4227	Non-antigen					
	HLA-B*27:05	RRAYAVYDL	35.3	0.6139	Antigen	Allergen				
		KRAEKVVSL	36.6	0.7044	Antigen	Allergen				
		KRGTVQTSL	102.4	-0.6252	Non-antigen					
	HLA-B*39:01	NKNAYSLYL	103.0	1.8591	Antigen	Allergen				
		KRAEKVVSL	168.7	0.7044	Antigen	Allergen				
		RRAYAVYDL	328.6	0.6139	Antigen	Allergen				
		FADVTTTSI	340.5	-0.5297	Non-antigen					
		LPITSSNEL	525.5	1.5083	Antigen	Allergen				
	HLA-B*58:01	LAAAVGAKW	3.6	1.2019	Antigen	Allergen				
		LSGNIVFNF	24.3	0.5158	Antigen	Allergen				
XP_002419306.1	HLA-A*01:01	VTSQYGHTY	103.8	1.2978	Antigen	Non-allergen	Non-Toxin		Inducer	No
		YTDKESIEV	109.0	1.6265	Antigen	Non-allergen	Non-Toxin		Inducer	No
		TSAIGIWGY	228.8	2.6174	Antigen	Allergen				
		NTYPFQIQY	450.1	2.2249	Antigen	Allergen				
		SSDLWVMSH	576.2	1.6375	Antigen	Allergen				
		LTNKQTFYM	626.2	-0.4471	Non-antigen					
		MLQSSSSSY	795.0	0.2578	Non-antigen					
	HLA-A*02:01	ILFGAIDHA	18.3	-1.4064	Non-antigen					
		YMLFGDNIL	18.6	1.0484	Antigen	Allergen				
		LLVAYCVLV	21.3	0.8605	Antigen	Allergen				
		ALLSLVATA	30.5	-0.8798	Non-antigen					
		NLDDYEVSL	34.7	1.7735	Antigen	Allergen				
	HLA-A*03:01	ALATTAPFK	16.0	0.0970	Non-antigen					
		HTYQNLPLK	27.4	-0.4843	Non-antigen					
		QTFYMATLK	27.7	-1.0773	Non-antigen					
		KIFGFGSVY	42.3	-1.2376	Non-antigen					
		HSAGSIIAK	128.6	0.7391	Antigen	Non-allergen	Non-Toxin		Inducer	No
	HLA-A*24:02	SYMLFGDNI	18.0	2.4644	Antigen	Allergen	Non-Toxin		Inducer	No
		TYSQISYPV	71.7	-0.1068	Non-antigen					
		TYQNLPLKL	73.8	-0.2858	Non-antigen					
		KYQGDLVTV	127.6	2.5881	Antigen	Non-allergen	Non-Toxin		Inducer	No

		QYGHTYQNL	204.8	-1.0397	Non-antigen					
		MYELLLSLL	219.3	-0.6592	Non-antigen					
	HLA-A*26:01	DTFKENNTY	72.7	-0.8235	Non-antigen					
		EVSLAQVSY	123.3	0.0927	Non-antigen					
		NTYPFQIQY	189.1	2.2249	Antigen	Allergen				
		YITEGPGAY	191.2	1.4188	Antigen	Allergen				
		YIVYNLDDY	413.9	1.3377	Antigen	Allergen				
		VTVKMMRTY	440.1	1.2038	Antigen	Non-allergen	Non-Toxin		Inducer	No
		ETSSDVGVL	466.2	2.1428	Antigen	Non-allergen	Non-Toxin		Inducer	
		ESIEVIGAS	646.7	1.6509	Antigen	Non-allergen	Non-Toxin		Inducer	No
		TSAIGIWGY	922.6	2.6174	Antigen	Allergen				
		EVIGASGIT	1112.8	0.9929	Antigen	Non-allergen	Non-Toxin		Non-Inducer	
	HLA-B*07:02	YPVRIQVPV	48.8	1.6273	Antigen	Non-allergen	Non-Toxin	Non-inducer	Inducer	Yes
	HLA-B*08:01	ELLQKRKNL	80.9	0.8448	Antigen	Non-allergen	Non-Toxin		Non-Inducer	
	HLA-B*27:05	MRLNSVALL	14.5	-0.3058	Non-antigen					
		GRSTCILGV	49.9	3.5563	Antigen	Non-allergen	Non-Toxin		Inducer	
	HLA-B*39:01	MRLNSVALL	39.8	-0.3058	Non-antigen					
		GHTYQNLPL	49.8	-1.2986	Non-antigen					
		FKENNTYPF	117.7	0.1848	Non-antigen					
		YMLFGDNIL	129.3	1.0484	Antigen	Allergen				
		YPVRIQVPV	235.9	1.6273	Antigen	Non-allergen	Non-Toxin	Non-inducer	Inducer	Yes
		SRSVGAYIV	302.1	2.4382	Antigen	Non-allergen	Non-Toxin		Inducer	No
		NLDDYEVSL	540.9	1.7735	Antigen	Allergen				
	HLA-B*40:01	IEFGGNKTI	30.4	3.0335	Antigen	Non-allergen	Non-Toxin		Inducer	No
		YELLLSLLV	58.6	-0.2083	Non-antigen					
		IEVIGASGI	79.6	-0.1658	Non-antigen					
	HLA-B*58:01	RSAYIVYNL	19.6	-0.4282	Non-antigen					
		SSSSSYMLF	24.0	1.3657	Antigen	Non-allergen	Non-Toxin	Non-inducer	Inducer	Yes
		MTLTNKQTF	46.8	-0.3736	Non-antigen					
		LATTAPFKI	99.3	0.5613	Antigen	Allergen				
XP_002420070.1	HLA-A*01:01	TSEDFSIGY	18.5	2.2932	Antigen	Non-allergen	Non-Toxin		Inducer	No
		TTEKDGQCY	68.4	-0.2177	Non-antigen					
		YSCTVNGTY	268.1	0.9381	Antigen	Allergen	Non-Toxin		Inducer	No
	HLA-A*02:01	YLAIMSNSV	6.8	0.0106	Non-antigen					
		FLLYLASVV	11.1	-1.6177	Non-antigen					
		ILFGAIDHA	18.3	-1.4064	Non-antigen					

	HLA-A*03:01	LLYLASVVK	32.4	-2.2039	Non-antigen					
		SVAPVAYTK	68.1	0.5889	Antigen	Allergen				
		MSNDAICYK	94.0	-0.5368	Non-antigen					
	HLA-A*24:02	GYDENFFGF	57.4	3.5318	Antigen	Non-allergen	Non-Toxin		Non-Inducer	
		VWGYDSVQF	77.7	-0.4405	Non-antigen					
		KYGVTDLKI	245.6	0.1226	Non-antigen					
	HLA-A*26:01	SAAQGVWGY	243.7	2.2386	Antigen	Allergen				
		DTGSTFSIF	347.6	1.6727	Antigen	Non-allergen	Non-Toxin		Inducer	No
		SVSIDTGSY	374.7	0.6273	Antigen	Allergen				
		NIVSTPSKY	680.3	0.9384	Antigen	Allergen				
		YTKRDALPM	830.3	-0.1044	Non-antigen					
		SVIGGGGIL	1000.4	0.7068	Antigen	Non-allergen	Non-Toxin		Non-Inducer	
	HLA-B*07:02	VPINSESQL	125.5	0.2740	Non-antigen					
	HLA-B*08:01	NMKKATSSA	158.3	1.7158	Antigen	Non-allergen	Non-Toxin		Inducer	No
		DILRRIYLV	217.7	0.0743	Non-antigen					
	HLA-B*27:05	RRIYLVYDL	13.6	1.9460	Antigen					
		KRDALPMPL	107.0	-0.3873	Non-antigen					
	HLA-B*39:01	FQTEGAPQL	21.4	-0.9693	Non-antigen					
		NHNEDQNEL	31.8	0.0011	Non-antigen					
		FPDEWIDAL	35.7	0.3194	Non-antigen					
		DHAKYDGAL	57.5	-1.1694	Non-antigen					
		NKIAYSVYL	58.2	0.5472	Antigen	Allergen				
		KRDALPMPL	88.4	-0.3873	Non-antigen					
		DKILYTTEL	196.6	-0.5927	Non-antigen					
		NKDKVSVSI	271.1	1.2857	Antigen	Allergen				
		NRSSVSDGI	411.1	0.4858	Non-antigen					
		RRIYLVYDL	442.6	1.9460	Antigen	Allergen				
		SKYTKRDAL	476.4	0.4425	Non-antigen					
	HLA-B*40:01	SESQLSVNV	86.2	1.9516	Antigen	Non-allergen	Non-Toxin		Inducer	No
		DENFFGFSI	177.2	2.1617	Antigen	Allergen				
		TEKDGQCYL	255.7	-0.2591	Non-antigen					
		SEDFSIGYV	260.2	1.7729	Antigen	Non-allergen	Non-Toxin	Non-inducer	Inducer	Yes
	HLA-B*58:01	CSIKLDFNI	72.2	3.3290	Antigen	Allergen				

Table S3: Protein ids with corresponding predicted B- cell epitopes with their corresponding calculated parameters

Protein ID	Peptide	Vaxijen score	Antigen /Non-antigen	Allergenicity	Toxicity	IL-2 inducer	IL-4 inducer	IFNepitope
>XP_002421073.1	PVNATGQDGKVKR	1.7534	Antigen	Non-allergen	Non-toxin	Inducer	Inducer	Yes
	DTGSSDLWI	0.4040	Non-antigen					
	SVTCENPPPGQSADFC	1.1736	Antigen	Allergen				
	GLYTPKSSTTSQRLG	0.9182	Antigen	Non-allergen	Non-toxin	Non-inducer		
	IGYGDGSSSHGTL	0.7310	Antigen	Non-allergen	Non-toxin	Inducer	Non-inducer	
	KTNEAAGDYDNVPV	0.3815	Non-antigen					
	LKQDGNGNS	1.1844	Antigen	Allergen				
>XP_002422286.1	TPKAFPVTNGQEGKTSKRQA	1.1864	Antigen	Non-allergen	Non-toxin	Inducer	Non-inducer	
	KIGYGDGSSSQGTLY	0.7588	Antigen	Non-allergen	Non-toxin	Non-inducer		
	YKTNEAGGDYDNVPV	0.1978	Non-antigen					
	ELTQDSSGN	1.5772	Antigen	Non-allergen	Non-toxin	Non-inducer		
>XP_002419429.1	PTTSKSKNSP	0.1599	Non-antigen					

	NVTGQQGNGKVTH	2.4219	Antigen	Non-allergen	Non-toxin	Non-inducer		
	DITVGSNNQ	1.5818	Antigen	Non-allergen	Non-toxin	Inducer	Non-inducer	
	SIEYGDGTTSQGTWYK	-0.0287	Non-antigen					
	FADVTSTSV	-0.0970	Non-antigen					
	QSHEAEGYYDN	-0.5299	Non-antigen					
>XP_002421072.1	TPVDQNDPTVRAK	1.1467	Antigen	Allergen				
	TGFPVDDTGRNDVVKR	-0.3337	Non-antigen					
	QKNEATESMY	0.6164	Antigen	Allergen				
	YTNGEPYPKC	-2.5867	Non-antigen					
	VKYTSQSDVVP	0.7560	Antigen					
>XP_002419185.1	AIPEDIDKR	0.8544	Antigen	Non-allergen	Non-toxin	Inducer	Inducer	No
	LINEGPSYAAT	-1.1039	Non-antigen					
	QYEDGSSSQGTVYK	0.2955	Non-antigen					
	TGDETNPTYDNVPV	0.1727	Non-antigen					

	TTVSASTTPVLDSG	0.2205	Non-antigen					
>XP_002419306.1	APSSKRHERSFGHG	0.9103	Antigen	Allergen				
	LYQPSKTIETDEEKDSSDK	0.5936	Antigen	Non-allergen	Non-toxin	Inducer	Inducer	Yes
	TEGPGAYST	1.4844	Antigen	Allergen				
	LEVTSQYGHT	1.0357	Antigen	Non-allergen	Non-toxin	Non-inducer		
	IDVEGSSGSTTN	2.9257	Antigen	Non-allergen	Non-toxin	Inducer	Non-inducer	
>XP_002420070.1	TPSKYTKRDA	0.5068	Antigen	Non-allergen	Non-toxin	Inducer	Non-inducer	
	EIGSNKDKVSVSIDTG	1.9489	Antigen	Non-allergen	Non-toxin	Non-inducer		
	FQTEGAPQLPD	-1.1836	Non-antigen					
	VDGSAAQGVWGY	2.1474	Antigen	Non-allergen	Non-toxin	Inducer	Inducer	No
	LETKDGKVTSG	2.7450	Antigen	Allergen				
	NATYDDNESV	-0.4621	Non-antigen					
	VAYTKDEDI	1.8338	Antigen	Allergen				

Supplementary Table S4. Physiochemical properties of vaccine candidate.

Number of amino acids	447
Molecular weight	45800.85
Antigenicity	Antigenic (Vaxijen score: 1.0662)
Allergic potential	Non-allergen
Theoretical pI	9.38
Instability index	38.93 (stable)
Total number of atoms	6419
Aliphatic index	83.56
Extinction coefficient	48930
Grand average of hydropathicity (GRAVY)	-0.172
Estimated half-life	30 hours (mammalian reticulocytes, in vitro). >20 hours (yeast, in vivo). >10 hours (*Escherichia coli*, in vivo).
